# *DNAH5* is associated with total lung capacity in chronic obstructive pulmonary disease

**DOI:** 10.1186/s12931-014-0097-y

**Published:** 2014-08-20

**Authors:** Jin Hwa Lee, Merry-Lynn N McDonald, Michael H Cho, Emily S Wan, Peter J Castaldi, Gary M Hunninghake, Nathaniel Marchetti, David A Lynch, James D Crapo, David A Lomas, Harvey O Coxson, Per S Bakke, Edwin K Silverman, Craig P Hersh, the COPDGene and ECLIPSE Investigators

**Affiliations:** Channing Division of Network Medicine, Brigham and Women’s Hospital, 181 Longwood Avenue, Boston, MA 02115 USA; Division of Pulmonary and Critical Care Medicine, Department of Internal Medicine, School of Medicine, Ewha Womans University, Seoul, South Korea; Division of Pulmonary and Critical Care, Brigham and Women’s Hospital, Boston, MA USA; Division of Pulmonary and Critical Care Medicine, Department of Medicine, Temple University School of Medicine, Philadelphia, PA USA; National Jewish Health, Denver, CO USA; Wolfson Institute for Biomedical Research, University College London, London, UK; Department of Radiology, University of British Columbia, Vancouver, Canada; Department of Clinical Science, University of Bergen, Bergen, Norway; Department of Thoracic Medicine, Haukeland University Hospital, Bergen, Norway

**Keywords:** Pulmonary disease, Chronic obstructive, Hyperinflation, Genome-wide association analysis, Total lung capacity, *DNAH5*

## Abstract

**Background:**

Chronic obstructive pulmonary disease (COPD) is characterized by expiratory flow limitation, causing air trapping and lung hyperinflation. Hyperinflation leads to reduced exercise tolerance and poor quality of life in COPD patients. Total lung capacity (TLC) is an indicator of hyperinflation particularly in subjects with moderate-to-severe airflow obstruction. The aim of our study was to identify genetic variants associated with TLC in COPD.

**Methods:**

We performed genome-wide association studies (GWASs) in white subjects from three cohorts: the COPDGene Study; the Evaluation of COPD Longitudinally to Identify Predictive Surrogate Endpoints (ECLIPSE); and GenKOLS (Bergen, Norway). All subjects were current or ex-smokers with at least moderate airflow obstruction, defined by a ratio of forced expiratory volume in 1 second to forced vital capacity (FEV_1_/FVC) <0.7 and FEV_1_ < 80% predicted on post-bronchodilator spirometry. TLC was calculated by using volumetric computed tomography scans at full inspiration (TLC_CT_). Genotyping in each cohort was completed, with statistical imputation of additional markers. To find genetic variants associated with TLC_CT_, linear regression models were used, with adjustment for age, sex, pack-years of smoking, height, and principal components for genetic ancestry. Results were summarized using fixed-effect meta-analysis.

**Results:**

Analysis of a total of 4,543 COPD subjects identified one genome-wide significant locus on chromosome 5p15.2 (rs114929486, β = 0.42L, *P* = 4.66 × 10^−8^).

**Conclusions:**

In COPD, TLC_CT_ was associated with a SNP in dynein, axonemal, heavy chain 5 (*DNAH5*), a gene in which genetic variants can cause primary ciliary dyskinesia. *DNAH5* could have an effect on hyperinflation in COPD.

**Electronic supplementary material:**

The online version of this article (doi:10.1186/s12931-014-0097-y) contains supplementary material, which is available to authorized users.

## Introduction

Chronic obstructive pulmonary disease (COPD) is one of the leading causes of morbidity and mortality worldwide [1]. Even though cigarette smoking is an established risk factor, only a minority of smokers develop COPD, which suggests that genetic susceptibility may play a significant role [2]. The pathophysiologic hallmark of COPD is expiratory flow limitation, resulting in air trapping and lung hyperinflation [3]. Dyspnea is the primary symptom limiting exercise in patients with advanced COPD. However, the physiologic impairment, traditionally measured by FEV_1_, often does not correlate strongly with the degree of dyspnea [4]. Hyperinflation at rest and during exercise is common among COPD patients and may be one explanation for differing exercise tolerance in patients with similar FEV_1_ levels [5]. Hyperinflation provides a mechanistic link between expiratory flow limitation and dyspnea [5]. Reducing hyperinflation is a mechanism for symptom relief from inhaled bronchodilators even in COPD patients who do not demonstrate a post-bronchodilator improvement in FEV_1_ [6]. Hyperinflation is closely linked to reduced exercise tolerance, poor quality of life, and even increased mortality in COPD subjects [7].

Genome-wide association studies (GWASs) of hyperinflation in COPD subjects have not been previously reported. We used total lung capacity measured by chest computed tomography scans (TLC_CT_) as an indicator of hyperinflation [8]. Our hypothesis was that genetic variants would be associated with TLC_CT_ in COPD.

## Methods

### Subjects

Subjects were current and former smokers from three COPD studies: the COPDGene Study (NCT00608764, http://www.copdgene.org); the Evaluation of COPD Longitudinally to Identify Predictive Surrogate Endpoints (ECLIPSE, NCT00292552, http://www.eclipse-copd.com); and GenKOLS (Bergen, Norway). All subjects had self-described European white ancestry, though African American subjects (AAs) from the COPDGene Study were included for an additional analysis. Study design and details of each study have been previously described [9–15]. Briefly, COPDGene is a multi-center genetic and epidemiologic investigation to study COPD. Subjects were between 45 and 80 years old and had at least 10 pack-years of cigarette smoking. ECLIPSE is a non-interventional, longitudinal prospective three-year study in cases with COPD and control subjects. The entry criteria for COPD cases and smoking controls were 40–75 years old with ≥10 pack-years of smoking. COPD was defined as FEV_1_ < 80% predicted and FEV_1_/FVC < 0.7. The control subjects were selected based on FEV_1_ ≥ 80% predicted and FEV_1_/FVC ≥ 0.7. In GenKOLS study, subjects for the case-control study were recruited from Bergen, Norway. Enrolment criteria were: 1) self-reported Caucasian; 2) aged > 40 years; 3) current or former smoker with ≥2.5 pack-years of smoking history; and 4) no severe α1-antitrypsin deficiency. The spirometry criteria were the same as ECLIPSE. Subjects with a history of lung volume reduction surgery were excluded from all three studies. The current analysis was approved by the Partners Healthcare Research Committee (COPDGene: 2007P000554; ECLIPSE: 2005P002467; GenKOLS: 2009P000790).

In this analysis, subjects with COPD were defined by having airflow obstruction of at least spirometry grade 2 (post-bronchodilator FEV_1_/FVC < 0.7 and FEV_1_ < 80% predicted), based on the Global initiative for chronic Obstructive Lung Disease (GOLD 2-4) [3]. Additional analyses included smokers with normal spirometry (post-bronchodilator FEV_1_/FVC ≥ 0.7 and FEV_1_ ≥ 80%).

### Chest CT scans

In each study, volumetric CT scans acquired in supine position at suspended full inspiration without administration of intravenous contrast. TLC_CT_ in liters was calculated from volumetric CT measurements.

In the COPDGene study, multi-detector CT scanners (at least 16 detector channels) were used. Detailed CT protocols have been previously published [10]. CT scans were subjected to a standard quality control procedure. Computerized image analysis was performed at the COPDGene Imaging Center at National Jewish Health and Brigham and Women’s Hospital using Slicer (Version 2, www.slicer.org). In the ECLIPSE study, multi-detector CT scanners (GE Healthcare or Siemens Healthcare) were used with a minimum of 4 detectors. Exposure settings were 120 kVp and 40 mAs and images were reconstructed at 1.0 mm (Siemens) or 1.25 mm (GE) contiguous slices, using a low spatial frequency reconstruction algorithm (GE: Standard, Siemens: b35f). CT scanners were calibrated regularly and a standard CT phantom was scanned by all participating centers to produce comparable data. All CT scans were analyzed using Pulmonary Workstation 2.0 software (VIDA Diagnostics, Iowa City, IA) [16]. In the GenKOLS study, a GE LightSpeed Ultra CT scanner (120 kVp, 200 mA; GE Healthcare, Milwaukee, WI, USA) was used with 1-mm slice thickness at 20-mm intervals. The CT scans were reconstructed using both a low spatial frequency reconstruction algorithm (standard) for density measurements, and a high spatial frequency algorithm (bone) for airway measurements. All ECLIPSE and GenKOLS images were transferred to the James Hogg iCAPTURE Centre (Vancouver, BC, Canada) for quantitative analysis as previously described [13].

### Genotyping quality control and imputation

Illumina platforms [HumanOmniExpress for COPDGene, HumanHap 550V3for ECLIPSE, and HumanHap 550 (V1, V3, and Duo) for GenKOLS; Illumina, Inc., San Diego, CA] were used for genotyping. Imputation in COPDGene was performed using MaCH [17] and minimac [18] using 1000 Genomes [19] Phase I v3 European (EUR) reference panels for non-Hispanic white subjects (NHWs). Details on genotyping quality control and imputation for the GenKOLS and ECLIPSE cohorts have been previously described [9,12,14,15,20,21]. Variants which passed genotyping or imputation quality control (R^2^ > 0.3) in all three cohorts, were included in the analysis.

### Statistical analysis

In each study population, we performed linear regression analysis of single nucleotide polymorphisms (SNPs) under an additive model of inheritance with adjustment for age, gender, height, pack-years of cigarette smoking and genetic ancestry-based principal components using PLINK 1.07 [22], as previously described [9,14,15]. Imputed genotypes were analyzed in a similar manner, using SNP dosage data in PLINK 1.07 [22]. Fixed-effects meta-analysis [23] was performed using METAL (version 2011-03-25) [24] and R 3.0.2 (www.r-project.org) with the meta-package. Genome-wide significance was determined by *P* value < 5 × 10^−8^. We evaluated heterogeneity by calculating both *I*^*2*^ [25] and *P* values for Cochrane’s *Q*. In regions with evidence of genetic heterogeneity, we also applied a modified random-effects model optimized to detect associations under heterogeneity since the fixed-effects model is based on inverse-variance-weighted effect size [26]. Genomic inflation factors [27] were calculated using GenABEL [28]. LocusZoom [29] was used to create local association plots, using the 1000 Genomes EUR reference data to calculate linkage disequilibrium (LD).

To explore other SNPs independently associated with TLC_CT_ in COPD, region-based conditional analyses were undertaken using linear regression with adjustment for the most significant (lead) SNP in a genome-wide significant region using genotyped or dosage data as appropriate. All SNPs within a 250 kb window on either side of the lead SNP were tested for association with TLC_CT_ in COPD. For region-based analyses conditional on the top SNP, a *P* value of *P* < 5 × 10^−4^ was considered significant to reflect an approximate adjustment for a 500 kb interval [9,15].

## Results

Baseline characteristics of each of the three cohorts are summarized in Table 1.

**Table 1:** **Baseline characteristics of COPD subjects included in the meta-analysis**

	**COPDGene**	**ECLIPSE**	**GenKOLS**
N	2,653	1,464	426
Age, yrs	64.7 (8.2)	63.5 (7.0)	64.3 (9.3)
Sex, male %	56.0	65.8	62.9
Current smoker, %	34.9	35.2	50.2
Pack-years of cigarette smoking	56.2 (27.9)	49.7 (26.7)	30.9 (18.2)
Height, cm	169.7 (9.4)	169.4 (9.0)	170.7 (8.7)
Body mass index, kg/m^2^	28 (6.1)	26.6 (5.6)	25.6 (4.8)
FEV_1_ % predicted	50 (17.9)	47.4 (15.5)	52.4 (17.0)
FVC % predicted	76.5 (17.0)	86.0 (19.9)	80.0 (15.3)
FEV_1_/FVC	0.49 (0.13)	0.44 (0.11)	0.52 (0.12)
Total lung capacity (TLC)_CT_, L	6.19 (1.4)	6.20 (1.44)	5.57 (1.29)
TLC % predicted	102.8 (16.6)	101.7 (18.2)	90.9 (18.8)

Data are presented as mean (SD) or percentage, as appropriate.

**Figure 1:**
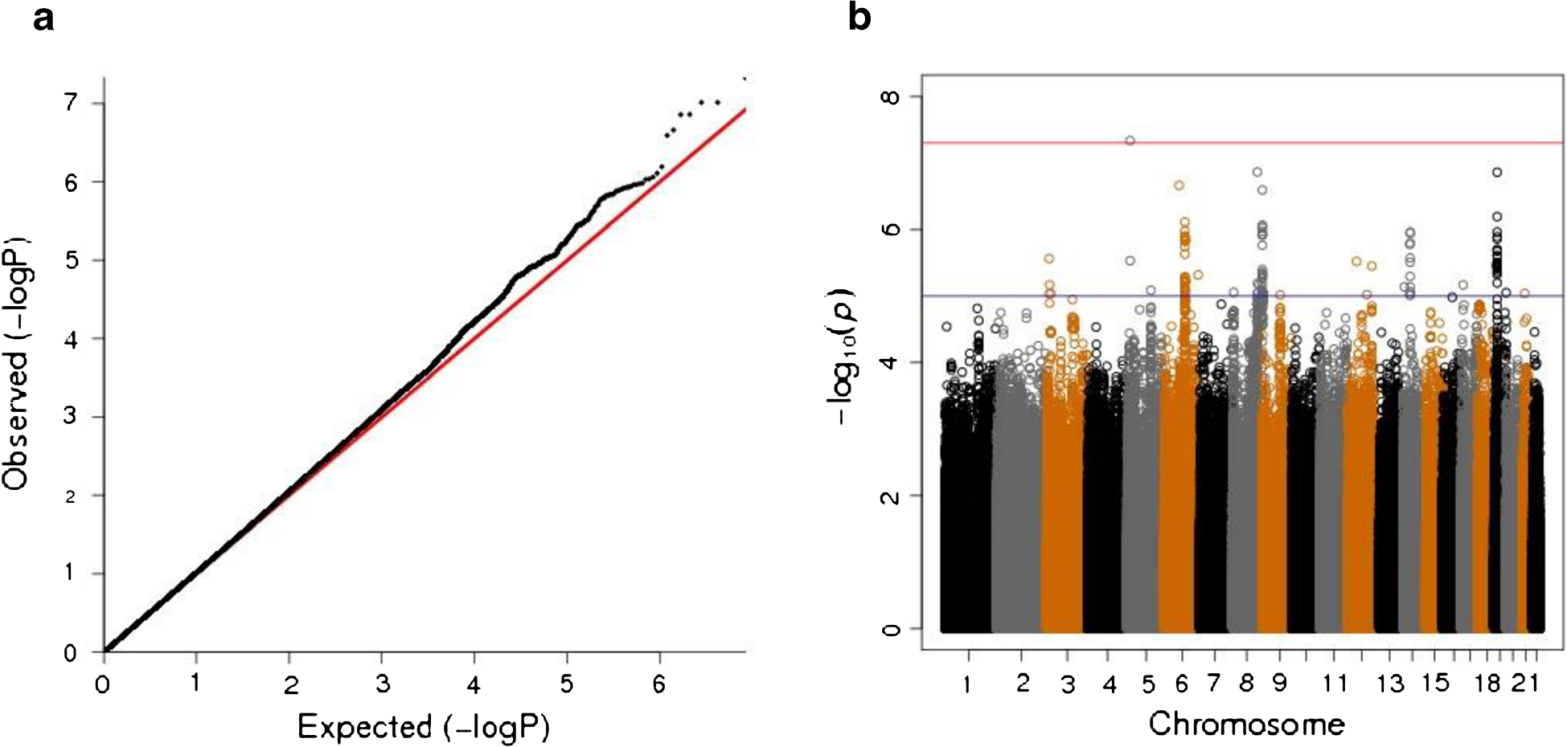
**The three-cohort meta-analysis including 1000 Genomes project imputed data for total lung capacity measured by chest CT in COPD subjects after adjustment for age, gender, height, pack-years of cigarette smoking and genetic ancestry-based principal components. (a)** The quantile–quantile plot and **(b)** Manhattan plot of –log10 P values.

**Figure 2:**
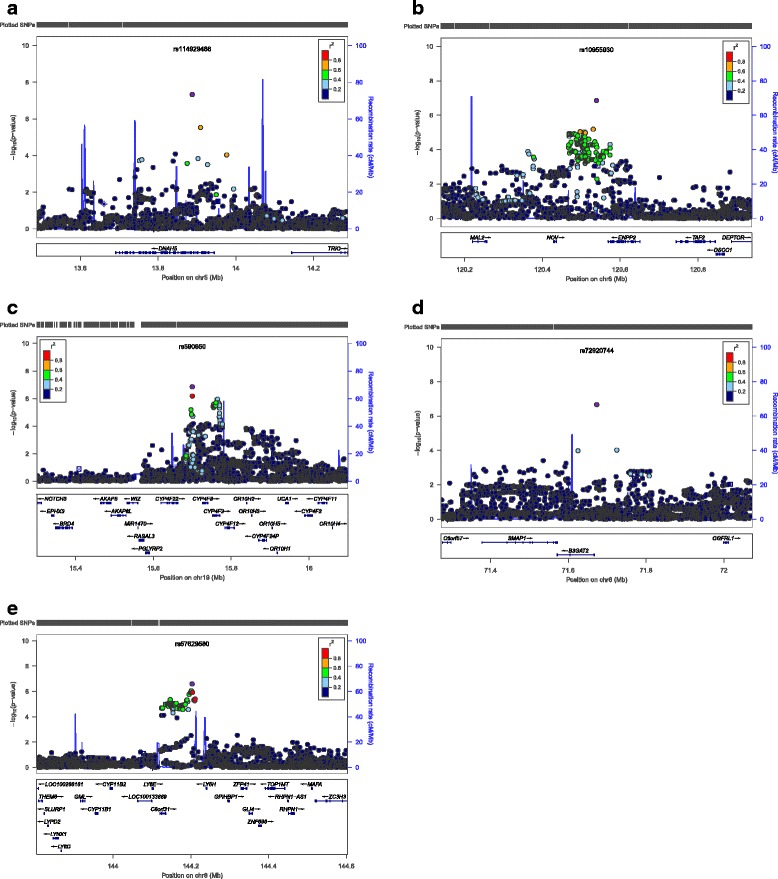
**The meta-analysis for total lung capacity measured by chest CT in COPD subjects.** Regional association plots for **(a)** one genome-wide significant locus and **(b-e)** the other four suggestive loci. The x-axis is chromosomal position, and the y-axis shows the -log10 P value. The most significant SNP at each locus is labeled in purple, with other SNPs colored by degress of linkage disequilibrium (r2). Plots were generated using LocusZoom.

In the meta-analysis of TLC_CT_ in COPD, the combined GWAS of three cohorts included 4,543 subjects with COPD. A quantile-quantile (Q-Q) plot is displayed in Figure 1A (lambda = 1.03). Figure 1B shows a novel region on chromosome 5p15.2, which reached the genome-wide significance threshold. Results yielding a suggestive *P* value threshold of < 5×10^−7^ [30] are listed in Table 2. Figure 2 displays the regional association plots for the top five loci.

**Table 2:** **Meta-analysis results of three genome-wide association studies for total lung capacity measured by CT in COPD subjects** ^*^

**Locus**	**SNP**	**Nearest Gene**	**Distance (kb)**	**Risk/Non-risk Allele**	**FRQ**	**COPDGene NHW**		**ECLIPSE**		**GenKOLS**		**Overall**		***I*** ^***2***^	***Q***
						**β**	***P***	**β**	***P***	**β**	***P***	**β**	***P***		
5p15.2	rs114929486	*DNAH5*	0	A/G	0.04	0.37^†^	2.94 × 10^−4^	0.60^†^	7.18 × 10^−6^	0.13^†^	5.47 × 10^−1^	0.42	4.66 × 10^−8^	49	0.14
8q24.12	rs10955930	*ENPP2*	30	T/A	0.49	0.11^†^	5.55 × 10^−4^	0.15^†^	1.19 × 10^−3^	0.22^†^	7.45 × 10^−3^	0.13	1.38 × 10^−7^	0	0.37
19p13.12	rs590950	*CYP4F8*	26	T/C	0.49	0.13^†^	3.36 × 10^−6^	0.09^†^	3.79 × 10^−2^	0.13^†^	1.25 × 10^−1^	0.12	1.39 × 10^−7^	0	0.72
6q13	rs72920744	*B3GAT2*	6	G/A	0.99	0.40^†^	8.05 × 10^−3^	0.83^†^	6.32 × 10^−6^	0.61^†^	1.11 × 10^−1^	0.58	2.18 × 10^−7^	39	0.19
8q24.3	rs57629580	*LY6H*	35	C/A	0.91	0.30^†^	1.47 × 10^−5^	0.27^†^	4.02 × 10^−3^	0.10^†^	6.05 × 10^−1^	0.28	2.56 × 10^−7^	0	0.66

Definition of abbreviations: FRQ = risk allele frequency; NHW = non-Hispanic white; SNP = single nucleotide polymorphism.

^*^Adjusted for age, sex, height, pack-years of cigarette smoking and genetic ancestry as summarized in the principal components.

^†^Imputed genotypes.

The most significant SNP on chromosome 5p15.2 was rs114929486 (β = 0.42L, *P =* 4.66 × 10^−8^), which was located within the gene dynein, axonemal, heavy chain 5 (*DNAH5*). Although some evidence of heterogeneity was present (*P* = 0.14 for Cochrane’s *Q*, *I*^*2*^ = −1.1), a modified random-effects meta-analysis model revealed similar significance (*P* = 6.15 × 10^−8^). The second most significant SNP was rs10955930 (β = 0.13L, *P* = 1.38 × 10^−7^), near ectonucleotide pyrophosphatase/phosphodiesterase 2 (*ENPP2*) on chromosome 8q24.12.

To determine whether there was likely to be more than one functional genetic variant within the genome-wide significant region, we performed analyses conditioning on the top (lead) SNP from the meta-analysis. All SNPs within 250 kb flanking the top signal were examined. We found evidence suggestive of secondary associations on 5p15.2 (conditioning on rs114929486) in two SNPs (rs4701985, β = 0.28L, *P* = 4.11 × 10^−4^; rs1502044, β = 0.31L, *P* = 4.45 × 10^−4^) located within the same gene, *DNAH5*.

### Additional analyses

There is significant difference in TLC_CT_ by genotypes of rs114929486 among COPD subjects of our study (Table 3). Additionally we compared clinical and radiological characteristics of the COPDGene NHW subjects stratified by genotypes of rs114929486 (Additional file 1: Table S1). Thicker airway walls (higher Pi10) and lower FEV_1_ % predicted values were seen among carriers of the risk allele for higher TLC (*P* <0.05).

**Table 3:** **Mean total lung capacity measured by CT (TLC**_**CT**_ **) according to genotype of rs114929486 in** ***DNAH5*** **(N = 4,543)** ^*^

	**rs114929486**	**GG**	**AG**	***P***
All	N	4282	258	
	TLC_CT_, L	6.12 (1.41)	6.50 (1.52)	0.0001
COPDGene	n	2531	121	
	TLC_CT_, L	6.18 (1.40)	6.47 (1.47)	0.042
ECLIPSE	N	1364	99	
	TLC_CT_, L	6.15 (1.42)	6.84 (1.57)	1.96 × 10^-5^
GenKOLS	N	387	38	
	TLC_CT_, L	5.56 (1.30)	5.70 (1.25)	0.486

Data are presented as mean (SD).

^*^Three subjects with AA genotype (each one from each cohort) were excluded from this analysis.

The top SNP, rs114929486, was very rare in African American subjects in the COPDGene Study (minor allele frequency = 0.0056) and therefore, was not analyzed further.

Hyperinflation may be due to different underlying mechanisms and genetic factors in different COPD patients, depending on their specific phenotypes. We performed additional GWASs and meta-analyses of TLC_CT_ in COPD subjects with either emphysema-predominant COPD (Additional file 1: Tables S2 and S4) or chronic bronchitis (Additional file 1: Tables S3 and S5). The stratified analyses did not show any genome-wide significant associations (Additional file 1: Figures S1 and S2). Among COPD subjects with ≥10% emphysema, chromosome 11p15.5, which includes genes *AP2A2*, *MUC6* and *CHID1*, was the most significant region (rs7483870, β = 0.16L, *P* = 3.93 × 10^−7^; rs4076950, β = 0.13L, *P* = 2.88 × 10^−6^; rs4963123, β = 0.13L, *P* =3.40 × 10^−6^). The top SNP from the original meta-analysis, rs114929486, located in *DNAH5* maintained nominal significance in both subjects with emphysema-predominant COPD (β = 0.39L, *P* = 4.19 × 10^−6^) and those with chronic bronchitis (β = 0.56L, *P* = 1.74 × 10^−4^).

We also performed meta-analyses of three GWASs for TLC_CT_ of smokers with normal spirometry (smoking controls) as well as combined analyses of both COPD and smoking control subjects. Additional file 1: Table S6 shows baseline characteristics of the included subjects. There were no genome-wide significant loci in the meta-analyses (Additional file 1: Figures S3 and S4) though three SNPs reached genome-wide significant thresholds in individual study GWAS. The results of the genome-wide significant SNPs were summarized in Additional file 1: Tables S7, S8 and S9.

## Discussion

This study is the first GWAS of total lung capacity in COPD subjects. Our meta-analysis of three GWASs of TLC_CT_ in subjects with moderate-to-severe COPD has found a genome-wide significant region on chromosome 5p15.2, containing the gene *DNAH5* and several suggestive loci on chromosomes 8q24, 19p13, and 6q13, with *P* values < 5 × 10^−7^.

The genome-wide significant SNP was located within *DNAH5*, encoding a dynein protein, which is part of a microtubule-associated motor protein complex consisting of heavy, light, and intermediate chains. This protein is an axonemal heavy chain dynein. It functions as a force-generating protein of respiratory cilia with ATPase activity, where the release of ADP is thought to produce the force-producing power stroke. Mutations in this gene can cause primary ciliary dyskinesia (PCD) type 3, as well as Kartagener syndrome, another disease due to ciliary defects [31–33]. PCD is characterized by marked peripheral airway dysfunction [34] and small airways obstruction leading to air trapping and a consequent increase in residual volume (RV) and RV/TLC ratio [35,36]. Similarly, the major sites of obstruction in COPD are small airways [37–39]. Recently a study demonstrated that cigarette smoking induces epithelial epigenetic changes in the small airway epithelium, collected by fiberoptic bronchoscopic brushings [40]. *DNAH5* was one of the most hyper-methylated genes in smokers compared to nonsmokers [40]. Therefore, *DNAH5* variants may contribute to genetic susceptibility to develop hyperinflation in patients with COPD.

The genome-wide significant SNP, rs114929486, and the other two significant SNPs from the region-based analysis conditional on the top SNP are located within intronic regions of *DNAH5*. These SNPs had minor allele frequency (MAF) of < 0.05 with relatively large effect sizes. In other diseases, an overlap between the genetic variants for Mendelian and complex diseases has been suggested [41,42]. Blair and colleagues [41] have found thousands of associations between Mendelian and complex diseases by mining the medical records of over 110 million patients. Using mathematical modeling, they also demonstrated that GWAS hits for common diseases are enriched in genes containing Mendelian variants [41]. Since the top GWAS SNP associated with TLC_CT_ is located in a gene for a Mendelian disorder, possible connections between *DNAH5* genetic variants and small airway disease should be investigated.

Although the second ranked SNP, rs10955930, did not reach genome-wide significance, the nearest gene, *ENPP2*, has been implicated in pulmonary fibrosis and allergic asthmatic inflammation [43]. The protein encoded by this gene, also known as autotaxin (ATX), functions as both a phosphodiesterase, which cleaves phosphodiester bonds at the 5′ end of oligonucleotides, and a phospholipase, which catalyzes production of lysophosphatidic acid (LPA) in extracellular fluids. LPA evokes growth factor-like responses including stimulation of cell proliferation and chemotaxis. ATX was highlighted as a therapeutic target for idiopathic pulmonary fibrosis since limiting LPA synthesis reduces fibrosis [44,45]. Recently, a study demonstrated marked and selective elevation of ATX and two of its LPA products, LPA 22:5 and LPA 22:6, in the bronchoalveolar lavage fluid of human patients with asthma in response to airway allergen challenge [46]. They also reported that ATX-overexpressing transgenic mice had a more severe asthmatic phenotype, whereas blocking ATX activity and knockdown of the LPA2 receptor in mice produced a marked attenuation of Th2 cytokines and allergic lung inflammation [46]. In the other study, *Enpp2*+/−mice, heterozygous for the autotaxin-encoding gene, showed not only diminished expression of autotaxin/lysophospholipase D and approximately half normal plasma LPA, but also exaggerated response to hypoxia-induced vasoconstriction and remodeling, as evidenced by increased right ventricular systolic pressure and an increased percentage of muscularized arterioles [47]. Even though there has been no direct evidence, *ENPP2* (ATX) could play a role in COPD-associated hyperinflation through its influence on extracellular matrix and inflammation.

The current study has several limitations. First, whether the associated regions play a functional role has not been investigated. Second, a replication analysis has not been performed in additional populations even though this was a meta-analysis of three GWASs using the largest COPD cohorts to date. Third, TLC is only one possible indicator of hyperinflation. There is no standard marker of hyperinflation and each indicator has its advantages and disadvantages. Besides TLC, RV, the ratio of RV/TLC, functional residual capacity (FRC), inspiratory capacity (IC) and the ratio of IC/TLC are all used to assess the severity of hyperinflation [8]. Most of these measures are obtained by helium dilution testing or plethysmography, which are challenging to implement in a large population study. TLC may be normal until the late stages of COPD and therefore may not be the best marker for hyperinflation in mild airflow limitation. However, our primary analysis was limited to subjects with moderate-to-severe airway obstruction. Fourth, TLC was measured by CT, not plethysmography, though strong correlations between TLC measured by plethysmography and CT have been observed [48–51]. However, in subjects with airflow limitation, plethysmography systematically overestimates lung volume relative to CT despite adherence to recommendations for proper measurement technique [52]. TLC_CT_ at full inspiration is a relatively standardized and widely used method [51] and may be easier for subjects to perform than plethysmography [52].

We have reported the first GWAS of TLC_CT_ in COPD subjects and identified *DNAH5* as a potential susceptibility gene associated with hyperinflation in COPD. The current study suggests that this gene for a Mendelian lung disease could also have an effect on hyperinflation in COPD, although future studies will be required for functional validation.

## Additional file

Additional file 1:
**Methods – Variable definitions. Table S1.** Clinical and radiological characteristics according to genotypes of rs114929486 among non-Hispanic white COPDGene subjects with COPD (GOLD 2-4) (N = 2,652). **Table S2.** Baseline characteristics of subjects with emphysema-predominant COPD. **Table S3.** Meta-analysis of total lung capacity measured by CT in emphysema-predominant COPD. **Table S4.** Baseline characteristics of COPD subjects with chronic bronchitis. **Table S5.** Meta-analysis results of total lung capacity measured by CT in COPD subjects with chronic bronchitis. **Table S6.** Baseline characteristics of COPD subjects and smokers with normal spirometry. **Table S7.** Results of a genome-wide association study (GWAS) for total lung capacity measured by CT in smokers with normal spirometry of ECLIPSE study. **Table S8.** Results of a GWAS for total lung capacity measured by CT in both of COPD subjects and smokers with normal spirometry of COPDGene Study. **Table S9.** Results of a GWAS for total lung capacity measured by CT in both of COPD subjects and smokers with normal spirometry of GenKOLS study. **Figure S1.** (A) The quantile–quantile plot and (B) Manhattan plot of –log10 *P* values for the three-cohort meta-analysis for total lung capacity measured by CT in COPD subjects with ≥10% emphysema. **Figure S2.** (A) The quantile–quantile plot and (B) Manhattan plot of –log10 *P* values for the three-cohort meta-analysis for total lung capacity measured by CT in COPD subjects with chronic bronchitis. **Figure S3.** (A) The quantile–quantile plot and (B) Manhattan plot of –log_10_ *P* values for the three-cohort meta-analysis for total lung capacity measured by CT in smokers with normal spirometry. **Figure S4.** (A) The quantile–quantile plot and (B) Manhattan plot of –log10 *P* values for the three-cohort meta-analysis for total lung capacity measured by CT in both of smokers with normal spirometry and COPD subjects.

## Abbreviations

AA: African American
ECLIPSE: Evaluation of COPD Longitudinally to Identify Predictive Surrogate Endpoints
GOLD: Global Initiative for chronic Obstructive Lung Disease
GWAS: Genome-wide association study
NHW: non-Hispanic white
SNP: Single nucleotide polymorphism
TLC: Total lung capacity
