# Supplementary material for: DNAH5 is associated with total lung capacity in chronic obstructive pulmonary disease
Source: Respir Res. 2014 Aug 20;15(1):97. doi: 10.1186/s12931-014-0097-y (PMC4169636; doi:10.1186/s12931-014-0097-y)

Methods

Variable definitions

In the COPDGene study, quantitative CT measurements were performed using Slicer (Version 2, www.slicer.org). Percent emphysema was calculated as the percentage of lung voxels with an attenuation < −950 Hounsfield units (HU) at full inspiration [% low attenuation area (LAA)-950_{insp}]. Airway analysis was performed using the Pulmonary Workstation Plus (VIDA Diagnostics, Inc., Coralville, IA). In each segmental bronchus, the wall area (WA) and lumen area (LA) were measured. The segmental wall area % was defined as 100 × WA/ (WA + LA). The square roots of the wall area for hypothetical airways with an internal perimeter of 10 mm (Pi10) and 15 mm (Pi15) were used as measures of airway disease. Gas trapping was quantified as the percentage of lung volume on expiratory CT with an attenuation < −856 HU (%LAA-856_{exp}).

Emphysema-predominant COPD was defined as ≥10% emphysema on chest CT scan. Chronic bronchitis was defined as chronic productive cough for 3 months in each of 2 successive years.

Table E1. Clinical and radiological characteristics according to genotypes of rs114929486 among non-Hispanic white COPDGene subjects with COPD (GOLD 2-4) (N=2,652)

Genotype	GG	AG	$P^{*,\dagger}$
n	2,531	121	
Age, years	64.7 (8.2)	65.0 (7.2)	NS
Female gender, %	55.9	58.7	NS
Pack-years of smoking	56.3 (27.9)	55.2 (27.3)	NS
Current smoker, %	34.7	39.7	NS
Height, cm	169.7 (9.3)	169.8 (9.9)	NS
Body mass index, kg/m^2	28.1 (6.1)	27.7 (6.8)	NS
6-min walked distance, feet	1,227 (388)	1,165 (373)	NS
Exacerbation frequency per yr	0.75 (1.24)	0.83 (1.18)	NS
Severe exacerbation, %	19.2	23.1	NS
Chronic bronchitis, %	29.9	30.6	NS
Total lung capacity $(TLC)_{CT}$, L	6.18 (1.40)	6.47 (1.47)	0.042
TLC % predicted	102.6 (16.5)	106.7 (17.3)	0.033
% emphysema	13.5 (12.8)	15.6 (13.9)	0.082
% emphysema upper third	15.9 (17.0)	18.6 (18.2)	0.067
% emphysema lower third	11.1 (11.1)	13.1 (12.6)	0.092
% emphysema upper 3rd/lower 3rd ratio	2.3 (5.2)	6.1 (43.0)	NS
% gas trapping	39.9 (20.5)	42.9 (20.6)	NS
Segmental wall area %	62.8 (3.0)	63.0 (3.3)	NS
Subsegmental wall area %	65.7 (2.4)	65.9 (2.6)	NS
Pi10	3.71 (0.14)	3.74 (0.16)	0.028
Pi15	5.21 (0.20)	5.22 (0.24)	NS
FEV_1 % predicted	50.2 (17.9)	46.4 (18.5)	0.025
FVC % predicted	76.6 (17.0)	75.5 (17.6)	NS
FEV_1/FVC	0.49 (0.13)	0.46 (0.14)	0.024
$FEF_{25-75\%}$, L/min	0.57 (0.36)	0.51 (0.30)	NS
Positive bronchodilator response, %	36.5	39.7	NS

Data are presented as mean (standard deviation) or percentage.
*Wilcoxon rank-sum test
†p-values <0.1 are expressed.
Severe exacerbation was defined as requiring emergency room visit or hospitalization in the year before study entry.
Bronchodilator responsiveness was considered positive if the change in FEV1 or FVC was ≥200mL and ≥ 12% predicted following administration of short-acting inhaled beta-agonist.
For 6-min walked distance, n = 2,571
For emphysema upper third, lower third, upper 3rd/lower 3rd ratio, n=2,610.
For % gas trapping, n = 2,449.
For segmental wall area %, n = 2,650.
For subsegmental wall area %, n =1,128.
For Pi10 and Pi15, n = 2,554.
For bronchodilator response, n = 2,638.

Table E2 Baseline characteristics of COPD subjects with emphysema-predominant COPD, defined as ≥10% emphysema (N=2,474)

	COPDGene	ECLIPSE	GenKOLS
N	1,279	1,020	175
Age, yrs	66.4 (7.3)	64.0 (6.6)	66.8 (8.4)
Sex, male %	58.5	67.5	68.0
Current smoker, %	19.7	27.9	41.7
Pack-years of cigarette smoking	59.0 (28.4)	50.5 (26.6)	33.7 (18.7)
Height, cm	169.6 (9.5)	169.5 (8.9)	170.1 (8.3)
Body mass index, kg/m^2	26.1 (5.0)	26.2 (5.5)	23.9 (3.9)
FEV_1 % predicted	40.9 (16.6)	43.8 (14.7)	41.2 (16.0)
FVC % predicted	75.4 (19.0)	86.6 (20.6)	77.7 (17.4)
FEV_1/FVC	0.40 (0.11)	0.40 (0.10)	0.42 (0.10)
Total lung capacity $(TLC)_{CT}$, L	6.66 (1.38)	6.58 (1.38)	6.35 (1.21)
TLC % predicted	110.3 (15.0)	107.5 (15.7)	103.1 (16.0)

Data are presented as mean (SD) or percentage, as appropriate.

Table E3 Meta-analysis of total lung capacity measured by CT in emphysema-predominant COPD *

Locus	SNP	Nearest Gene	Distance (kb)	Risk/Non -risk Allele	FRQ	COPDGene NHW		ECLIPSE		GenKOLS		Overall		I^2	Q
						β	*P*	β	*P*	β	*P*	β	*P*		
11p15.5	rs7483870	*AP2A2*	0	G/A	0.21	0.18	7.42×10^{-6}	0.09	9.03×10^{-2}	0.29	1.71×10^{-2}	0.16	3.93×10^{-7}	-25	0.20
6q22.31	rs55927589	*PKIB*	0	C/A	0.04	0.51†	5.11×10^{-5}	0.38†	1.31×10^{-2}	0.60†	9.21×10^{-2}	0.47	5.69×10^{-7}	0	0.76
3p26.1	rs1356229	*SUMF1*	0	C/T	0.97	0.32†	1.96×10^{-3}	0.55†	4.10×10^{-5}	-0.15†	7.36×10^{-1}	0.39	1.38×10^{-6}	-16.9	0.18
11p15.5	rs4076950	*MUC6*	0	C/T	0.33	0.12	8.86×10^{-4}	0.11	1.60×10^{-2}	0.27	7.90×10^{-3}	0.13	2.88×10^{-6}	-76.4	0.32
7q35	rs28702020	*CNTNAP2*	0	G/A	0.18	0.13†	3.46×10^{-3}	0.17†	3.88×10^{-3}	0.54†	1.80×10^{-3}	0.16	3.18×10^{-6}	25.3	0.07
11p15.5	rs4963123	*CHID1*	4	T/C	0.56	0.14†	2.48×10^{-5}	0.04†	4.16×10^{-1}	0.40†	1.07×10^{-3}	0.13	3.40×10^{-6}	50.3	0.02
16p12.2	rs78091431	*USP31*	0	C/T	0.98	0.43†	3.29×10^{-4}	0.42†	1.36×10^{-2}	0.51†	1.21×10^{-1}	0.43	3.64×10^{-6}	0	0.97
2q32.3	rs13035689	*PCGEM1*	16	C/A	0.05	0.25	7.83×10^{-4}	0.26	8.97×10^{-3}	0.53	3.85×10^{-2}	0.27	3.72×10^{-6}	0	0.57
5p15.2	rs114929486	*DNAH5*	0	A/G	0.04	0.39†	1.66×10^{-3}	0.52†	8.33×10^{-5}	-0.07†	7.77×10^{-1}	0.39	4.19×10^{-6}	10.1	0.11

Definition of abbreviations: *AP2A2* = adaptor-related protein complex 2, alpha 2 subunit; *CHID1* = chitinase domain containing 1; *CNTNAP2* = contactin associated protein-like 2; *DNAH5* = dynein, axonemal, heavy chain 5; FRQ = risk allele frequency; *MUC6* = mucin 6, oligomeric mucus/gel-forming; *PCGEM1* = PCGEM1, prostate-specific transcript (non-protein coding); *PKIB* = Protein Kinase (CAMP-Dependent, Catalytic) Inhibitor Beta; SNP = single nucleotide polymorphism; *SUMF1* = sulfatase modifying factor 1; *USP31* = ubiquitin specific peptidase 31.

*Adjusted for age, sex, height, pack-years of cigarette smoking and genetic ancestry as summarized in the principal components.

†Imputed genotypes

Table E4 Baseline characteristics of COPD subjects with chronic bronchitis (N=1,340)

	COPDGene	ECLIPSE	GenKOLS
N	795	391	154
Age, yrs	62.8 (8.3)	62.3 (7.5)	64.4 (9.3)
Sex, male %	62.6	73.1	65.6
Current smoker, %	53.2	51.4	56.5
Pack-years of cigarette smoking	59.3 (28.4)	51.9 (30.7)	32.3 (20.4)
Height, cm	170.3 (9.3)	170.4 (8.8)	170.8 (8.9)
Body mass index, kg/m^2	27.9 (6.2)	26.0 (5.3)	25.4 (4.9)
FEV_1 % predicted	49.0 (17.4)	46.1 (15.3)	47.5 (16.1)
FVC % predicted	76.3 (17.9)	84.5 (19.7)	77.7 (16.1)
FEV_1/FVC	0.48 (0.13)	0.44 (0.12)	0.49 (0.12)
Total lung capacity $(TLC)_{CT}$, L	6.41 (1.44)	6.37 (1.46)	5.83 (1.34)
TLC % predicted	104.4 (17.1)	102.0 (18.9)	94.3 (17.8)

Data are presented as mean (SD) or percentage, as appropriate.

Table E5 Meta-analysis of total lung capacity measured by CT in COPD subjects with chronic bronchitis*

Locus	SNP	Nearest Gene	Distance (kb)	Risk/Non-risk Allele	FRQ	COPDGene NHW		ECLIPSE		GenKOLS		Overall		I^2	*Q*
						β	*P*	β	*P*	β	*P*	β	*P*		
18q22.2	rs4891832	*RTTN*	41	C/T	0.74	0.25	1.39×10^{-5}	0.28	6.48×10^{-3}	0.07	6.36×10^{-1}	0.24	4.40×10^{-7}	0	0.51
11q24.3	rs12805375	*APLP2*	6	T/C	0.05	0.40†	8.66×10^{-4}	0.25†	2.16×10^{-1}	1.20†	1.09×10^{-5}	0.47	9.94×10^{-7}	79.2	0.01
11q24.3	rs56052964	*ST14*	4	T/C	0.05	0.38†	1.38×10^{-3}	0.25†	2.20×10^{-1}	1.20†	1.19×10^{-5}	0.46	1.78×10^{-6}	79.3	0.01
1p21.1	rs77583284	*OLFM3*	0	G/A	0.94	0.43†	9.67×10^{-5}	0.55†	7.91×10^{-3}	0.24†	4.25×10^{-1}	0.44	2.22×10^{-6}	0	0.70
11p15.1	rs1715301	*NELL1*	0	T/G	0.78	0.47†	2.29×10^{-5}	0.32†	9.39×10^{-2}	0.35†	1.66×10^{-1}	0.42	2.23×10^{-6}	0	0.77
10q23.1	rs12775803	*GRID1-AS1*	270	A/T	0.05	0.42†	5.40×10^{-4}	0.52†	8.69×10^{-3}	0.40†	1.11×10^{-1}	0.43	3.84×10^{-6}	0	0.88

Definition of abbreviations: *APLP2* = amyloid beta (A4) precursor-like protein 2; FRQ = risk allele frequency; *GRID1-AS1* = GRID1 antisense RNA 1; *NELL1* = NEL-like 1 (chicken); *OLFM3* = olfactomedin 3; *RTTN* = rotatin; SNP = single nucleotide polymorphism; *ST14* = suppression of tumorigenicity 14 (colon carcinoma).

*Adjusted for age, sex, height, pack-years of cigarette smoking and genetic ancestry as summarized in the principal components.

†Imputed genotypes

Table E6 Baseline characteristics of COPD cases and smoking controls*

	COPDGene		ECLIPSE		GenKOLS	
	Controls	COPD and Controls	Controls	COPD and Controls	Controls	COPD and Controls
N	2,397	5,050	151	1615	409	835
Age, yrs	59.5 (8.6)	62.3 (8.8)	57.3 (9.3)	62.9 (7.5)	55.7 (9.4)	60.1 (10.3)
Sex, male %	48.9	52.6	58.9	65.2	53.3	58.2
Current smoker, %	39.3	37.0	40.7	35.7	40.3	45.4
Pack-years of cigarette smoking	37.9 (20.1)	47.5 (26.1)	31.9 (26.2)	48.0 (27.2)	19.8 (14.1)	25.5 (17.2)
Height, cm	169.7 (9.4)	169.7 (9.4)	171.6 (9.6)	169.6 (9.1)	172.3 (8.5)	171.5 (8.6)
BMI, kg/m^2	28.9 (5.6)	28.5 (5.9)	27.2 (4.3)	26.7 (5.5)	26.5 (4.1)	26.0 (4.5)
FEV_1 % predicted	96.8 (11.0)	72.2 (27.8)	108.5 (13.2)	53.0 (23.4)	94.9 (9.2)	73.2 (25.3)
FVC % predicted	95.4 (11.2)	85.5 (17.4)	111.9 (14.8)	88.4 (20.9)	97.9 (9.6)	88.8 (15.6)
FEV_1/FVC	0.78 (0.05)	0.63 (0.18)	0.79 (0.05)	0.44 (0.55)	0.79 (0.04)	0.65 (0.16)
TLC_{CT}, L	5.67 (1.25)	5.94 (1.36)	5.18 (1.36)	6.11 (1.47)	4.39 (1.13)	4.99 (1.35)
TLC_{CT} % predicted	95.4 (14.0)	99.3 (15.8)	83.7 (17.2)	100.0 (18.8)	71.3 (16.3)	81.3 (20.2)

Data are presented as mean (SD) or percentage, as appropriate.
Definition of abbreviations: BMI = body mass index; TLC_{CT} = total lung capacity measured by computed tomography.
*Controls were defined as smokers with post-bronchodilator FEV_1/FVC ≥0.7 and FEV_1 ≥80% predicted. COPD was defined as smokers with post-bronchodilator FEV_1/FVC < 0.7 and FEV_1 <80% predicted.

Table E7 Results of a genome-wide association study for total lung capacity measured by CT in smokers with normal spirometry of ECLIPSE study*

Locus	SNP	Nearest Gene	Risk/Non-risk Allele	FRQ	β	*P*
9q33.1	rs7852026	*DBC1*	C/T	0.874	1.00†	3.85×10^{-8}
9q33.1	rs7850456	*DBC1*	G/A	0.86	0.87†	3.73×10^{-7}
9q33.1	rs10984095	*DBC1*	C/G	0.86	0.84†	4.40×10^{-7}

Definition of abbreviations: *DBC1* = deleted in bladder cancer 1; FRQ = risk allele frequency; SNP = single nucleotide polymorphism.

*Adjusted for age, sex, height, pack-years of cigarette smoking and genetic ancestry as summarized in the principal components.

†Imputed genotypes

Table E8 Results of a genome-wide association study for total lung capacity measured by CT in both of COPD subjects and smokers with normal spirometry of COPDGene Study*

Locus	SNP	Nearest Gene	Risk/Non-risk Allele	FRQ	β	*P*
4q28.3	rs112314153	*PABPC4L*	C/T	0.07	0.22†	3.45×10^{-8}
4q28.3	rs17577653	*PABPC4L*	A/G	0.07	0.21†	1.12×10^{-7}

Definition of abbreviations: *PABPC4L* = poly (A) binding protein, cytoplasmic 4-like; FRQ = risk allele frequency; SNP = single nucleotide polymorphism.

*Adjusted for age, sex, height, pack-years of cigarette smoking and genetic ancestry as summarized in the principal components.

†Imputed genotypes

Table E9 Results of a genome-wide association study for total lung capacity measured by CT in both of COPD subjects and smokers with normal spirometry of GenKOLS study*

Locus	SNP	Nearest Gene	Risk/Non-risk Allele	FRQ	β	*P*
14q23.1	rs8015490	*DAAM1*	A/G	0.93	0.63†	1.75×10^{-8}
14q23.1	rs77459056	*DACT1*	T/C	0.93	0.58†	3.52×10^{-7}
14q23.1	rs77830478	*DACT1*	T/C	0.93	0.58†	3.52×10^{-7}

Definition of abbreviations: *DAAM1* = dishevelled associated activator of morphogenesis 1; *DACT1* = dishevelled-binding antagonist of beta-catenin 1; FRQ = risk allele frequency; SNP = single nucleotide polymorphism.

*Adjusted for age, sex, height, pack-years of cigarette smoking and genetic ancestry as summarized in the principal components.

†Imputed genotypes

Figure E1 (A) The quantile–quantile plot and (B) Manhattan plot of –log10 *P* values for the three-cohort meta-analysis including 1000 Genomes project imputed genotype data for total lung capacity measured by chest CT in COPD subjects with ≥10% emphysema after adjustment for age, gender, height, pack-years of cigarette smoking and genetic ancestry-based principal components.

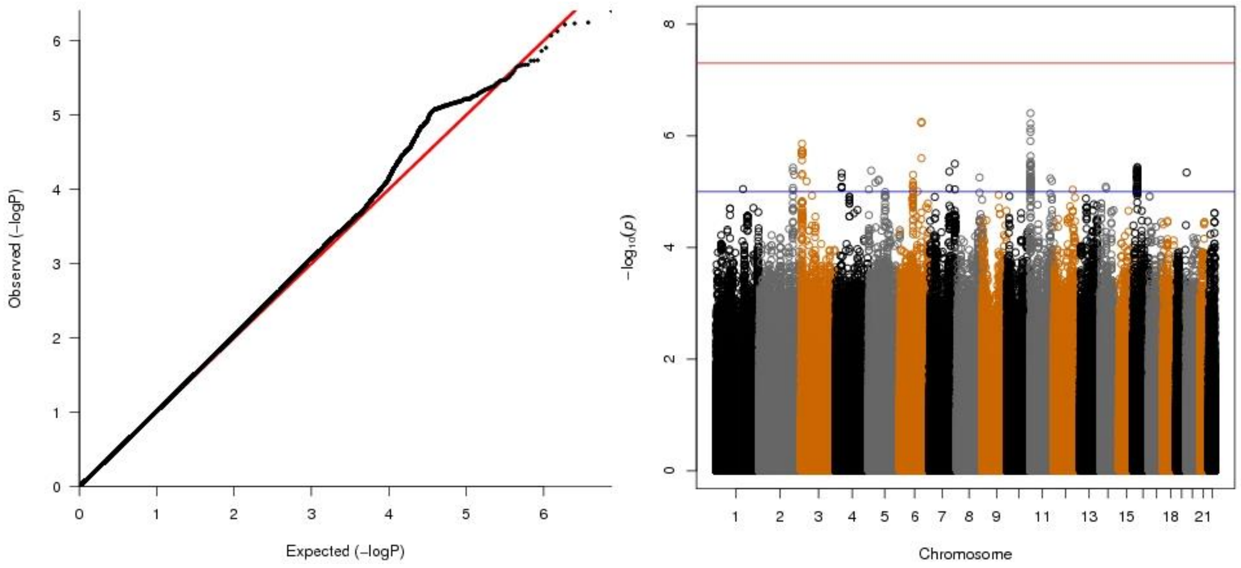

Figure E2 (A) The quantile–quantile plot and (B) Manhattan plot of –log10 *P* values for the three-cohort meta-analysis including 1000 Genomes project imputed genotype data for total lung capacity measured by chest CT in COPD subjects with chronic bronchitis after adjustment for age, gender, height, pack-years of cigarette smoking and genetic ancestry-based principal components.

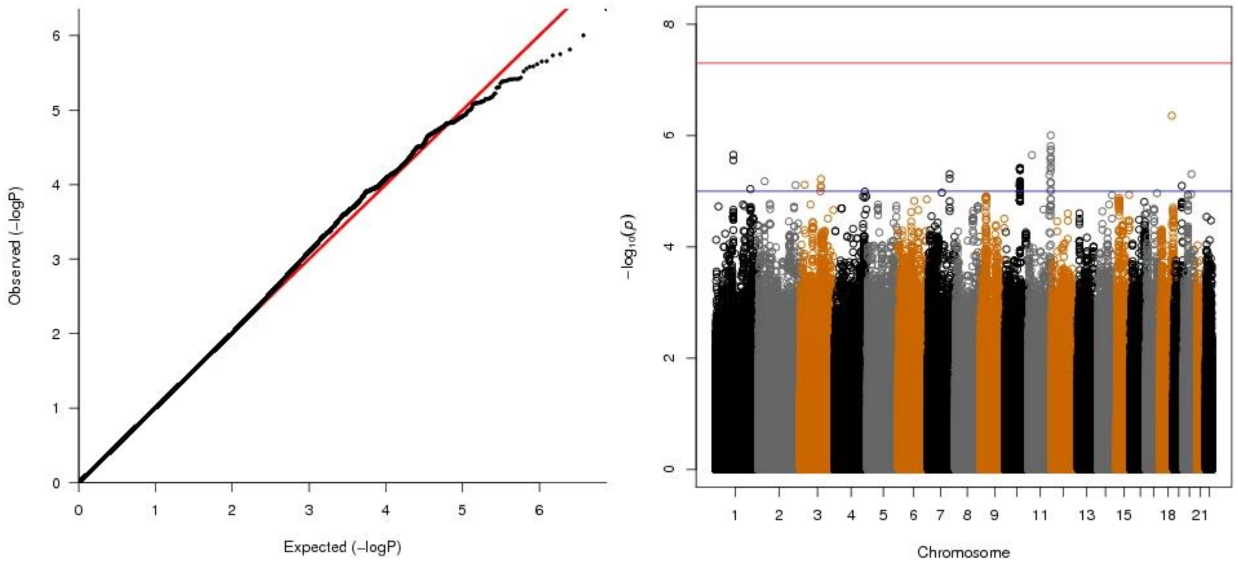

Figure E3 (A) The quantile–quantile plot and (B) Manhattan plot of $–\log_{10} P$ values for the three-cohort meta-analysis including 1000 Genomes project imputed genotype data for total lung capacity measured by chest CT in smokers with normal spirometry after adjustment for age, gender, height, pack-years of cigarette smoking and genetic ancestry-based principal components.

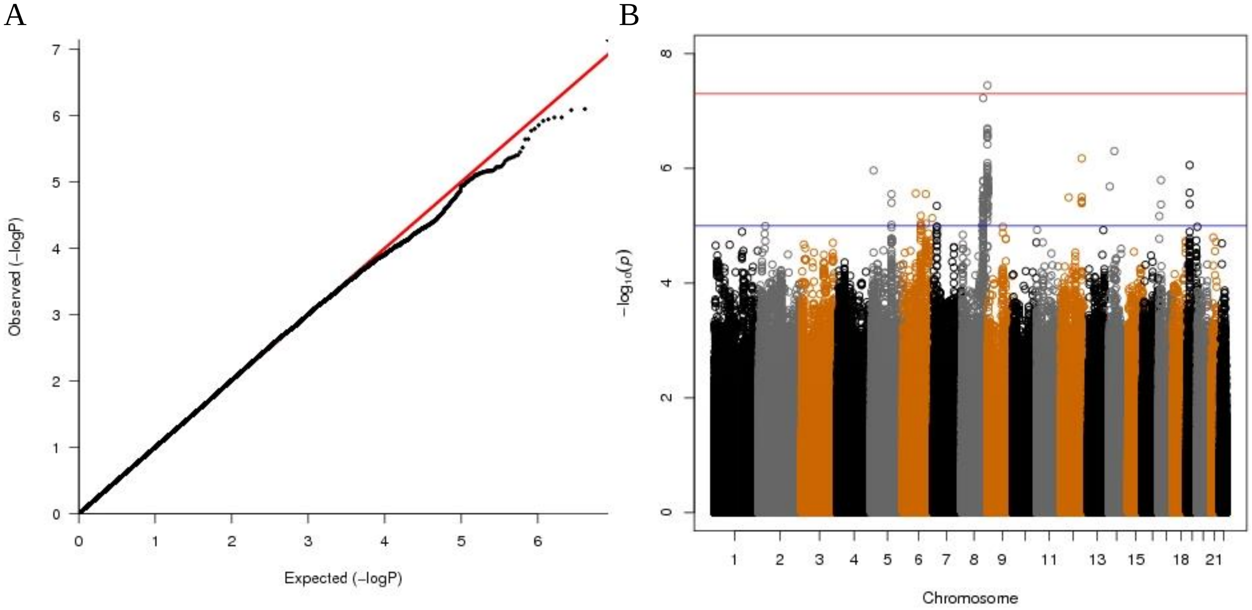

Figure E4 (A) The quantile–quantile plot and (B) Manhattan plot of –log10 *P* values for the three-cohort meta-analysis including 1000 Genomes project imputed genotype data for total lung capacity measured by chest CT in both of smokers with normal spirometry and COPD subjects after adjustment for age, gender, height, pack-years of cigarette smoking and genetic ancestry-based principal components.

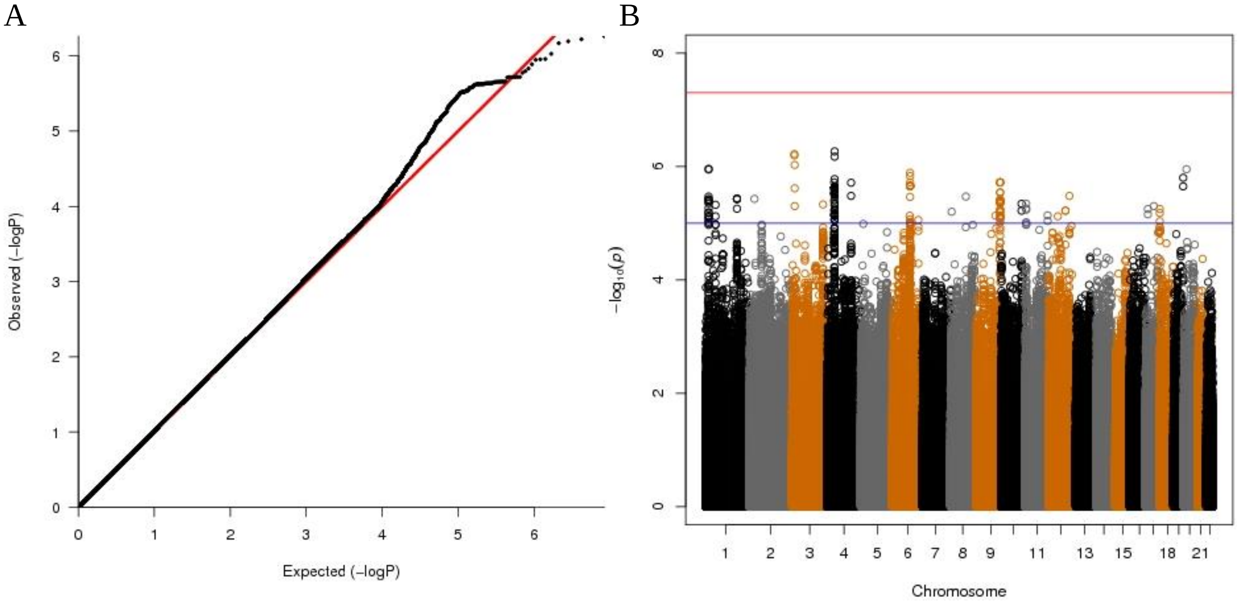

Supplement: Additional file 1: — Methods – Variable definitions. Table S1. Clinical and radiological characteristics according to genotypes of rs114929486 among non-Hispanic white COPDGene subjects with COPD (GOLD 2-4) (N = 2,652). Table S2. Baseline characteristics of subjects with emphysema-predominant COPD. Table S3. Meta-analysis of total lung capacity measured by CT in emphysema-predominant COPD. Table S4. Baseline characteristics of COPD subjects with chronic bronchitis. Table S5. Meta-analysis results of total lung capacity measured by CT in COPD subjects with chronic bronchitis. Table S6. Baseline characteristics of COPD subjects and smokers with normal spirometry. Table S7. Results of a genome-wide association study (GWAS) for total lung capacity measured by CT in smokers with normal spirometry of ECLIPSE study. Table S8. Results of a GWAS for total lung capacity measured by CT in both of COPD subjects and smokers with normal spirometry of COPDGene Study. Table S9. Results of a GWAS for total lung capacity measured by CT in both of COPD subjects and smokers with normal spirometry of GenKOLS study. Figure S1. (A) The quantile–quantile plot and (B) Manhattan plot of –log10 P values for the three-cohort meta-analysis for total lung capacity measured by CT in COPD subjects with ≥10% emphysema. Figure S2. (A) The quantile–quantile plot and (B) Manhattan plot of –log10 P values for the three-cohort meta-analysis for total lung capacity measured by CT in COPD subjects with chronic bronchitis. Figure S3. (A) The quantile–quantile plot and (B) Manhattan plot of –log10 P values for the three-cohort meta-analysis for total lung capacity measured by CT in smokers with normal spirometry. Figure S4. (A) The quantile–quantile plot and (B) Manhattan plot of –log10 P values for the three-cohort meta-analysis for total lung capacity measured by CT in both of smokers with normal spirometry and COPD subjects. [file 12931_2014_97_MOESM1_ESM.pdf]
